# Motorcycle-related trauma:effects of age and site of injuries on mortality. A single-center, retrospective study.

**DOI:** 10.1186/s13017-020-00297-1

**Published:** 2020-03-10

**Authors:** Stefano S. Granieri, Elisa E. Reitano, Francesca F. Bindi, Federica F. Renzi, Fabrizio F. Sammartano, Stefania S. Cimbanassi, Shailvi S. Gupta, Osvaldo O. Chiara

**Affiliations:** 1 General Surgery and Trauma Team, ASST Niguarda, Milano, University of Milan, Piazza Ospedale Maggiore 3, 20162 Milan, Italy; 2 Adams Cowley Shock Trauma Center, University of Maryland, Baltimore, MD USA

**Keywords:** Motorcycle, Motorcycle crash, Trauma

## Abstract

**Background:**

Motorcyclists are often victims of road traffic incidents. Though elderly patients seem to have worse survival outcomes and sustain more severe injuries than younger patients, concordance in the literature for this does not exist. The aim of the study is to evaluate the impact of age and injury severity on the mortality of patients undergoing motorcycle trauma.

**Methods:**

Data of 1725 patients consecutively admitted to our Trauma Center were selected from 2002 to 2016 and retrospectively analyzed. The sample was divided into three age groups: ≤ 17 years, 18–54 years, and ≥ 55 years. Mortality rates were analyzed for the overall population and patients with Injury Severity Score (ISS) ≥ 25. Differences in survival among age groups were evaluated with log-rank test, and multivariate logistic regression models were created to identify independent predictors of mortality.

**Results:**

A lower survival rate was detected in patients older than 55 years (83.6% vs 94.7%, *p* = 0.049) and in those sustaining critical injuries (ISS ≥ 25, 61% vs 83%, *p* = 0.021). Age (*p* = 0.027, OR 1.03), ISS (*p* < 0.001, OR 1.09), and Revised Trauma Score (RTS) (*p* < 0.001, OR 0.47) resulted as independent predictors of death. Multivariate analysis identified head (*p* < 0.001, OR 2.04), chest (*p* < 0.001, OR 1.54), abdominal (*p* < 0.001, OR 1.37), and pelvic (*p* = 0.014, OR 1.26) injuries as independent risk factors related to mortality as well. Compared to the theoretical probability of survival, patients of all age groups showed a survival advantage when managed at a level I trauma center.

**Conclusions:**

We detected anatomical injury distributions and mortality rates among three age groups. Patients aging more than 55 years had an increased risk of death, with a prevalence of severe chest injuries, while younger patients sustained more severe head trauma. Age represented an independent predictor of death. Management of these patients at a level I trauma center may lead to improved outcomes.

## Background

Motorcyclists represent a significant percentage of road traffic victims worldwide and have a greater risk of injury-related death than car occupants [1, 2]. Despite the burden of injuries associated with motorcycle incidents, few comprehensive studies have been conducted to examine the outcome of these patients according to age and site of injuries. Identifying high-risk injury patterns and common clusters of ages may allow for targeted interventions leading to improved care [3, 4].

Generally, young motorcyclists are more likely to be involved in incidents under the influence of alcohol, riding without insurance, or not wearing a helmet or other protective clothing [5]. Furthermore, a significant correlation was found between risk perception and traffic condition awareness for experienced drivers [6]. Given previous studies, a driver’s age and experience are used worldwide as components of licensing restrictions to help reduce the burden of injuries from road traffic incidents [7].

Results reported in the literature regarding mortality rates between younger and older motorcyclists and the patterns of injuries in different age groups are often discordant. Some authors suggest that advanced age may be associated with worse outcomes after motorcycle trauma whereas other studies suggest the opposite [7–13]. The reason for this disagreement may lie in the different cut-offs given by various authors in order to define the “elderly” group. Furthermore, advanced age is associated with changes in physiology and chronic diseases and drugs can alter the body response to injury, lowering the threshold of tolerance. From the other side, the old and experienced motorcycle driver is more prudent because of the awareness of traffic-related hazards. Given the discordance in the current literature, the aim of this study was to evaluate the correlation between the severity of injuries stratified by anatomical region and age with mortality rates of patients involved in motorcycle incidents managed at a level I trauma center.

## Methods

All details about trauma patients managed at Niguarda Hospital, a level 1 Trauma Center in Milan, Italy [14], are collected in the Niguarda trauma registry, in which demographic data, mechanism of trauma, pre-hospital and in-hospital clinical conditions, diagnostic and therapeutic procedures, and survival outcome are recorded. The registry is held by a Trauma Team consultant who is meant to keep it constantly updated, and it is annually revised by the head of the department. All motorcycle-related consecutive incidents from 2002 to 2016 were selected from the registry and demographic data, and abbreviated injury scale (AIS, 1998 version) score of each anatomical region, Injury Severity Score (ISS), Revised Trauma Score (RTS), and probability of survival (PS) obtained by Trauma and Injury Severity Score (TRISS) system, length of hospitalization, and survival outcome were retrospectively analyzed. The American Society of Anesthesiologists (ASA) physical status classification was chosen to summarize comorbidities in an ordinal fashion; unfortunately, these data have been available only from 2011 onwards. Injuries were grouped by anatomical region: head, face, chest, abdomen, pelvis, extremities, and external according to AIS classification. Patients were divided into three age groups: ≤ 17 years, 18–54 years, and ≥ 55 years. The 17-year-old age cut-off was selected according to the Italian legislation definition of legal age, whereas the 55 years cut-off was identified based on the TRISS calculator age coefficient turning point.

Data were recorded in a computerized spreadsheet (Microsoft Excel 2016; Microsoft Corporation, Redmond; WA) and analyzed with statistical software (IBM Corp., released 2012, IBM SPSS Statistics for Windows, Version 21.0; Armonk, NY, IBM Corp.).

The sample distribution was evaluated with Kolmogorov-Smirnov and Shapiro-Wilk tests resulting in a non-Gaussian distribution for any of the examined variable. Continuous variables were compared by independent sample Kruskal-Wallis test, while categorical variables were analyzed using Pearson’s chi-squared test. Mortality rates were obtained for the overall population and patients with ISS ≥ 25 considering the age group stratification mentioned above. In-hospital mortality was estimated calculating the elapsed time since admission to the Emergency Room to hospital discharge or death.

Survival curves were obtained with Kaplan-Meier analysis, and log-rank test was assessed to evaluate differences in cumulative survival among age groups. Bivariate logistic regression was used to provide odds ratio for individual variables, identifying possible predictors of mortality. Two different multivariate regression models were then built: one for general variables (age, ISS, RTS, PS) and another one for injured anatomical regions in order to detect independent risk factors for death and to estimate the adjusted odds ratio (OR) and 95% confidence interval (CI). Only significant (*p* < 0.1) variables at bivariate analysis were included in the multivariate models *p* values below .05 were considered statistically significant.

We then investigated the efficacy of providing care at a level I trauma center by comparing the observed survival rate with the estimated one, obtained by the Trauma Injury Severity Score (TRISS) system.

The institution of trauma registry for all major trauma admitted to our trauma center has been approved by the Niguarda Ethical Committee Milano Area 3 (record number 534-102018). Given the retrospective nature of the study, a specific ethical review board approval was not required.

## Results

During a 14-year period, from 2002 to 2016, 6691 major trauma patients were managed at Niguarda Hospital, a level 1 Trauma Center in Milan, Italy. A total of 1725 motorcycle incident victims were selected from the trauma registry. One hundred thirty-four patients (7.8%) were less than 18 years old (group 1), 1447 (83.9%) between 18 and 54 years old (group 2), and 144 (8.3%) more than 55 years old (group 3). Of the overall population, 26.8% (*n* = 462) sustained critical injuries, as defined by an ISS ≥ 25.

Further clinical characteristics of the study population are summarized in Table 1.

**Table 1:** Demographic- and trauma-related data and comparison among age groups

Variables	Age groups (%)				*p*
	≤ 17 (134)	18–54 (1447)	≥ 55 (144)	Total (1725)	
Gender					0.054
Male	114 (85.1)	1303 (90)	135 (93.8)	1552 (90)	
Female	20 (14.9)	144 (10)	9 (6.3)	173 (10)	
Age					
Mean	15.6	34	61	34.85	
S.D.	2.2	9.9	5.8	13.12	
Outcome					0.049**
Survived	128 (95.5)	1382 (95.5)	131 (91)	1641 (95.1)	
Dead	6 (4.5)	65 (4.5)	13 (9)	84 (4.9)	
ASA***					< 0.001*
1	51 (100)	724 (88.5)	61 (55.5)	836 (85.4)	
2	0	90 (11)	41 (37.3)	131 (13.4)	
3	0	4 (0.5)	8 (7.3)	12 (1.2)	
ISS					0.662
Mean	16.34	16.96	18.22	17.02	
S.D.	13.07	14.44	16.12	14.48	
RTS					0.702
Mean	7.23	7.16	7.07	7.16	
S.D.	1.47	1.52	1.87	1.55	
Probability of survival					
Mean	91.44%	90.97%	81.46%	90.21%	< 0.001*
S.D.	19.09	21.1	29.29	21.91	
Length of hospitalization (days)					0.648
Mean	15.9	17.04	14.85	16.76	
S.D.	20.28	22.67	17.81	22.13	

*ASA* American Society of Anesthesiologists, *ISS* Injury Severity Score, *RTS* Revised Trauma Score, *TRISS* Trauma and Injury Severity Score

*Significant value

**Log-rank test

***Data available only for 979 patients

No differences among age groups were noticed regarding ISS, RTS, and length of hospital stay, but mortality was double (9% vs 4.5%) in the older age group as compared to the other groups.

Since we started collecting comorbidities information from 2011 onward, ASA score was available only for 979 patients. More than 85% of this subset of patients had an ASA score of 1, and patients belonging to the third group had the greatest proportion of ASA 2 and 3. The comparison among groups showed a significant difference (*p* < 0.001). Probability of survival estimated with Trauma and Injury Severity Score (TRISS) was 91.44% ± 19.09 for group 1, 90.97% ± 21.1 for group 2, and 81.46% ± 29.39 for group 3 (*p* < 0.001).

**Fig. 1:**
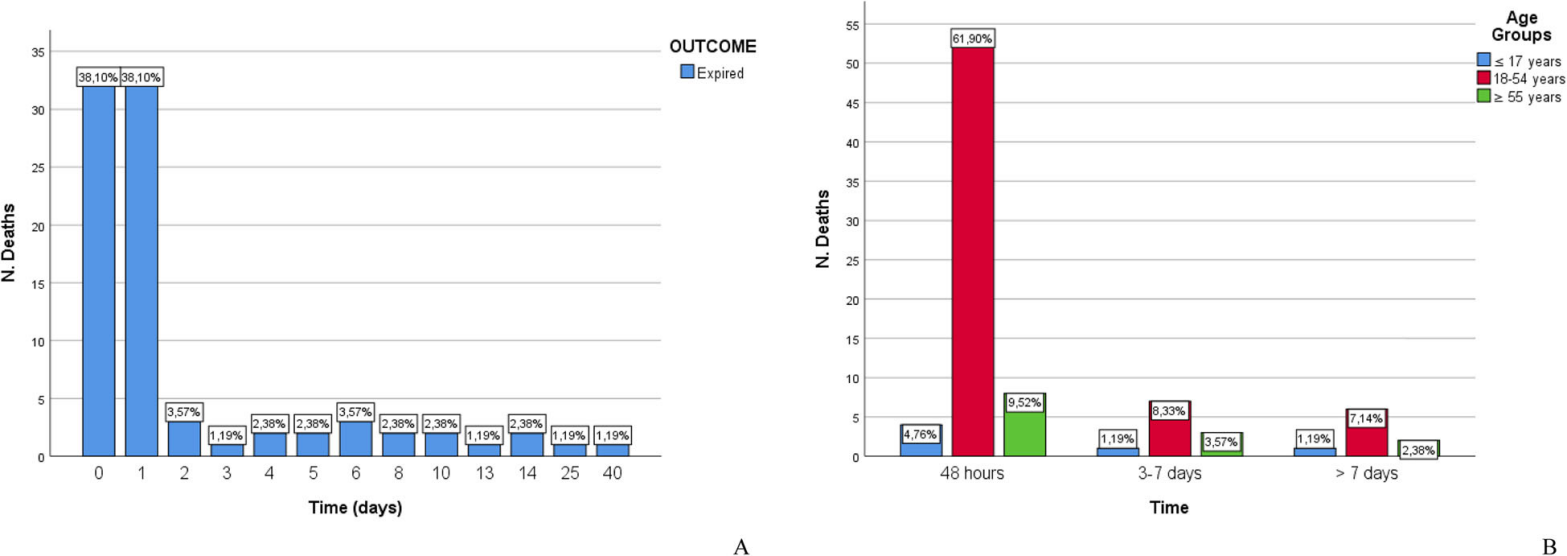
**a**) Distribution of deaths over time; **b**) Distribution of deaths among age groups in different time clusters (acute - within 48 hours; early - from 3 to 7 days; late - beyond 7 days).

The mortality rate in the overall population was 4.9% (84/1725) (Table 1). More than three fourths of the patients (64/84, 76.2%) died in the first 48 h and almost 62% of them belonged to the second group (18–54 years). Figure 1 shows mortality trend over time (A) and mortality distribution among age groups according to a previous study published by our group (B) [15].

**Fig. 2:**
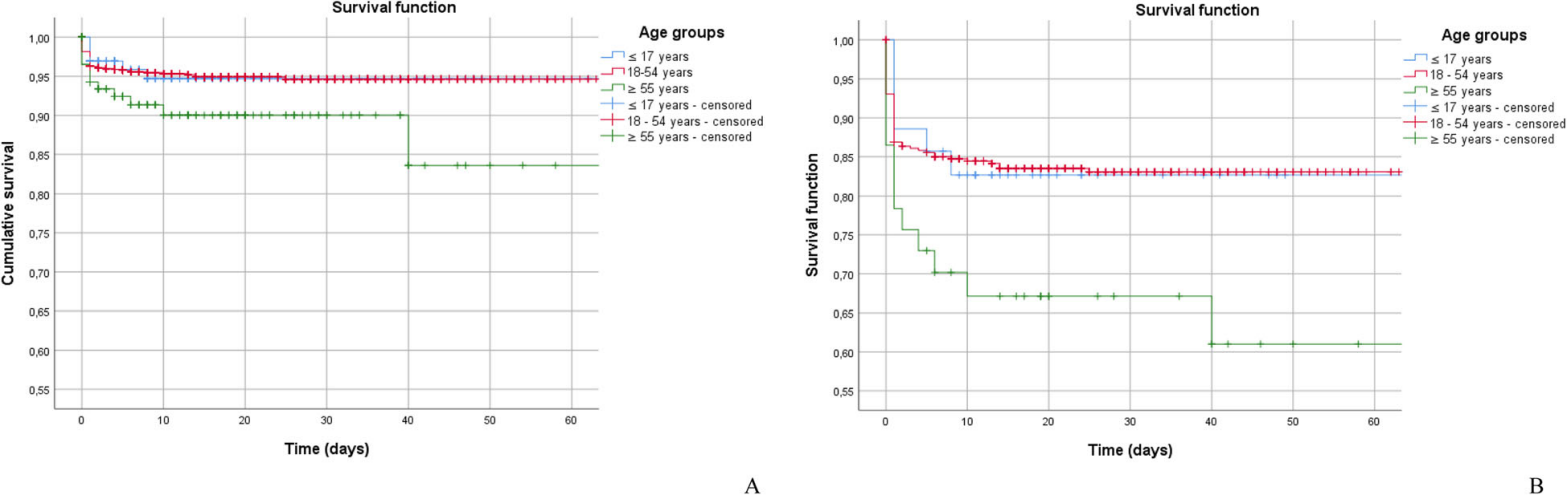
**a**) Survival trand of the overall population stratified according to age; **b**) Survival trend of the subgroup of patients sustaining critical injuries (ISS ≥ 25) stratified according to age.

The survival rate computed with Kaplan-Meier method was 99.9% in patients with an ISS ≤ 24 and 80.9% in more severely injured patients, with ISS ≥ 25 (log-rank test, *p* < 0.001). Moreover, survival was different in the three age groups. Patients aging more than 55 years showed a significantly worse prognosis compared with younger patients (log-rank test: *p* = 0.049, Fig. 2a). The reduction in survival was more evident for patients older than 55 years old with ISS ≥ 25 (log-rank test: *p* = 0.021, Fig. 2b), with a striking drop during the first 10 days after trauma.

The overall distribution of injuries stratified by anatomical district is reported in Table 2.

**Table 2:** Anatomical districts of injuries and anatomical districts of injuries AIS 98′ ≥ 3

Age groups (%)				
Anatomical districts of injuries	≤ 17 (134)	18–54 (1447)	≥ 55 (144)	Total (1725)
Head	85 (28.6)	758 (22.2)	85 (22.3)	928 (22.65)
Face	22 (7.4)	280 (8.2)	31 (8.2)	333 (8.12)
Chest	30 (10)	532 (15.6)	73 (19.1)	635 (15.49)
Abdomen	25 (8.4)	332 (9.7)	34 (8.9)	391 (9.54)
Pelvis	13 (4.4)	184 (5.4)	22 (5.8)	219 (5.34)
Extremities	60 (20.1)	730 (21.3)	74 (19.4)	864 (21.08)
Surface	63 (21.1)	602 (17.6)	62 (16.3)	727 (17.74)
Anatomical districts of injuries AIS 98′ ≥ 3	≤ 17	18–54	≥ 55	Total
Head	44 (37.3)	300 (22.3)	36 (24.2)	380 (23.59)
Face	1 (0.8)	30 (2.2)	3 (2)	34 (2.11)
Chest	20 (17)	458 (34.1)	56 (37.6)	534 (33.15)
Abdomen	19 (16.1)	204 (15.2)	15 (10)	238 (1477)
Pelvis	9 (7.6)	124 (9.2)	17 (11.4)	150 (9.31)
Extremities	25 (21.2)	227 (16.9)	22 (14.8)	274 (17.01)
Surface	–	1 (0.1)	–	1 (0.06)

Head, extremities, and external injuries were the most common, whereas in critical injuries defined by an AIS 98′ ≥ 3, chest, head, and extremity injuries were the most common. Torso injuries (chest, abdomen, and pelvis together) were 30.4% in overall population and 57.2% when only AIS ≥ 3 were considered. Severe head injuries were prevailing in younger (37.3%) and chest injuries less represented (17%). In older patients, the opposite result was observed: critical chest injuries were more frequent (37.6%) than head injuries (24.2%).

Significant variables which correlated with mortality using bivariate logistic regression analysis were ISS, RTS, and PS. Because of substantial multicollinearity with age (variance inflation factor (VIF) 6.31), PS was excluded from the multivariate model. All variables entered in the multiple regression model were confirmed independent predictors of death (Table 3). A subgroup analysis was conducted to evaluate the effect of comorbidities (using ASA classification) on the outcome. Bivariate logistic regression failed to demonstrate a potential predicting role of comorbidities on survival (*p* = 0.358, OR 0.52, 95% CI, 0.129–2.095)

**Table 3:** Bivariate and multivariate analysis: general variables

Bivariate analysis: general variables				
	*p*	OR	CI 95%	
			Lower	Upper
Male Gender	0.109	2.296	0.831	6.347
Age	0.06	1.016	0.999	1.032
ISS	< 0.001*	1.136	1.113	1.159
RTS	< 0.001*	0.363	0.314	0.419
PS (TRISS)	< 0.001*	0.001	0.001	0.002
Multivariate analysis: general variables				
	*p*	Adjusted OR	CI 95%	
			Lower	Upper
Age	0.027*	1.03	1.003	1.057
ISS	< 0.001*	1.091	1.064	1.118
RTS	< 0.001*	0.472	0.399	0.558

*ISS* Injury Severity Score, *RTS* Revised Trauma Score, *TRISS* Trauma and Injury Severity Score

*Significant value

Similarly, districts of injury were individually evaluated with bivariate logistic regression which pointed out a significant correlation with mortality for all of them. A second multivariate analysis was then realized, showing that the head, chest, abdominal, and pelvic injuries were all independent risk factors related to mortality, whereas extremity injuries were associated with improved survival. Considering only severe injuries (AIS 98′ ≥ 3), multivariate analysis demonstrated that head, chest, and abdominal injuries were independent risk factors related to mortality, with elevated odds ratios particularly for head and chest injuries (Table 4).

**Table 4:** Multivariate analysis: injured districts predictors of death in whole population. Multivariate analysis: injured districts AIS 98′ ≥ 3 predictors of death in whole population

	*p*	Adjusted OR	CI 95%	
			Lower	Upper
Multivariate analysis: injured districts predictors of death in whole population				
Head	< 0.001*	2.036	1.749	2.37
Face	0.197	1.189	0.914	1.547
Chest	< 0.001*	1.546	1.337	1.788
Abdomen	< 0.001*	1.373	1.171	1.61
Pelvis	0.014*	1.257	1.047	1.511
Extremities	0.003*	0.715	0.574	0.89
Multivariate analysis: injured districts AIS 98′ ≥ 3 predictors of death in whole population				
Head	< 0.001*	8.792	5.295	14.599
Face	0.072	2.568	0.92	7.169
Chest	< 0.001*	3.684	2.202	6.164
Abdomen	0.001*	2.432	1.415	4.18
Pelvis	0.174	1.637	0.805	3.329

*AIS* Abbreviated Injury Scale 1998

*Significant value

By comparing observed and TRISS-estimated survival rates, patients of all age groups treated at a trauma center had a survival advantage. The result was even more relevant when considering the subgroup of patients with ISS ≥ 25 and patients older of 55 years (Table 5).

**Table 5:** Observed and estimated survival rates

Age groups			
Overall survival rate	≤ 17	18–54	≥ 55
Observed	94.7%	94.7%	83.6%
Estimated (TRISS)	91.4%	90.9%	81.5%
Absolute difference	3.2%	3.8%	2.1%
Survival rate for ISS ≥ 25	≤ 17	18–54	≥ 55
Observed	82.7%	83%	61%
Estimated (TRISS)	74.8%	71.2%	43.1%
Absolute difference	7.9%	11.8%	17.9%

*TRISS* Trauma and Injury Severity Score

## Discussion

Motorcyclists represent a quarter of road deaths in the world and a consistent part of all traffic victims. The number of motorcyclists suffering from road trauma is growing due to the rapid global expansion of the motorcycle market. The use of motorcycles is expanding also in older ages for enhanced mobility in heavy-traffic urban areas. Some evidences suggest that age is generally an important predictor of mortality related to traumatic events [16, 17]. Our data demonstrate that older patients had an increased mortality for severe injuries. Head, chest, abdominal, and pelvic injuries were all independent predictors of death; severe head injuries occurred more frequently in younger patients, while chest injuries were more common in older patients.

The relationship between age, the severity of injuries, and mortality following motorcycle trauma is still controversial [8, 13]. Mullin et al. demonstrated a relationship of inverse proportionality between age with risk of death and severe injuries in motorcyclists and car drivers [7]. Increased age of motorcycle drivers has been strongly highlighted as a protective factor against fatal and non-fatal injuries deriving from motorcycle crashes [8, 18], due to the greater driving experience of older patients. Other studies showed that the elderly population have a higher risk of severe injuries and death [19]. Underlying diseases in the older population would increase mortality for all types of trauma [20].

Some investigators suggest that drivers older than 40 years are 25% more prone to death after motorcycle injuries [12]. Moreover, Richter et al. compared crash injury rates between older and younger road users, detecting a higher severity of the injuries and mortality rate in the older cohort [19].

In the present study, the overall mortality rate was 4.9% for all motorcycle injuries, with the highest rate among the older group (≥ 55 years) and multivariate analysis confirmed that age is an independent predictor of death.

ASA score was available just for a bit more than a half of our sample. Due to this limitation, we could not adjust our survival analysis for comorbidities. Anyway, the subgroup analysis failed to highlight a contribution of comorbidities on the survival outcome and the negative OR of bivariate logistic regression would indicate a worse outcome for lower ASA scores. This could be explained by the fact that all ASA 3 patients survived; by contrast, 22 out of 24 deaths in the subgroup were ASA 1.

Despite the results of the subgroup analysis, because of the similar values of ISS and RTS between age groups, we tend to believe that older patients have a lower tolerance for injuries of the same severity and increased ASA score may be a determinant of worsen outcome.

Talving et al. focused on the anatomical region injured, underlining that older patients, defined as greater than 55 years old, are significantly more likely to suffer severe head injuries, chest injuries, and spinal fractures [1]. Dischinger et al. demonstrated that motorcyclists older than 40 years old show a significantly higher incidence of multiple thoracic injuries [21].

In our investigation, head injuries were the anatomic region most frequently injured overall. In the subset of severe injuries, chest injuries (33.1%) and head injuries (23.6%) were the most represented. By considering the group of critical injuries defined by an AIS 98′ ≥ 3, patients sustaining head trauma had a near 9-fold increased risk of death, whereas those sustaining chest and abdominal injuries had a 3.6-fold and a 2.4-fold increased mortality risk, respectively. Older ages were associated with higher mortality and with a higher frequency of chest injuries with less severe head injuries. The thoracic cage of the elderly is more prone to costal and sternal fractures resulting in severe injuries to internal organs, which may be fatal. Given the atrophy of the brain in elderly patients, severe head injuries may evolve more slowly, as more blood is required to cause increased intracranial pressure. Younger patients have less atrophy, and thus, an even small bleed may progress to clinically significant increased intracranial pressure.

It is worth noting that on multivariate analysis, extremity injuries showed a correlation with a hypothetical improved prognosis (OR 0.715, 95% CI 0.574–0.89). This effect could be explained by the high number of extremity injuries, present in approximately 20% of the overall population, the majority of whom have survived.

In our sample mortality distributed in a bimodal fashion with a greater proportion of acute (within the first 48 h) rather than early or late deaths confirms the findings of other authors in the current literature [22–26]. The same trend was observed after stratifying the sample according to age, although in the older group the difference between acute and early/late mortality was less remarkable.

Many data available in the literature demonstrate improved survival of major trauma patients when treated in a dedicated trauma center showing a reduction in mortality rate, length of hospital stay, and an improved physical function [27–30]. MacKenzie et al. demonstrated besides that the overall risk of death for trauma injuries is significantly lower when care is provided in a trauma center [31]. Although TRISS calculation has been largely questioned, it is still the most prominent method for trauma care benchmarking, and the comparison between expected and observed survival is a good way to measure efficacy of care. Our data confirm the benefits of dedicated care at a trauma center and highlight an important survival benefit, more evident in severely injured and older patients. This underlines the importance of a dedicated team composed of physicians and nurses skilled in the management of trauma.

To our knowledge, our study represents the largest single-center representation of major motorcycle injuries at a level 1 trauma center in Europe with the review of 1725 patient’s data collected in a standardized registry during a 14-year period.

Nevertheless, some limitations exist in our study. A major concern could be raised considering that our data are not adequately weighted: especially in the older group, the influence of comorbidities on prognosis cannot be neglected. However, this type of data has started being gathered in the registry only since 2011, so our decision to not control for comorbidities is due to a lack of information in the database that eventually could alter the accuracy of the results. Finally, due to the non-homogeneous distribution of the sample among age groups (with most of the study population belonging to the young adults’ group), the opportunity to investigate the effect of every anatomical region injured in each age group was precluded. Moreover, it is possible that a potential survivorship bias comparing young, adults, and senior patients exists because in our database, information about pre-hospital are unavailable due to an increase of the pre-hospital mortality.

Moreover, the Trauma and Injury Severity Score (TRISS) was originally conceived in 1983 and in 2010, and its coefficients were further revised [32, 33]. This scoring system is based on data obtained from North American trauma registries, the American College of Surgeons Committee on Trauma National Trauma Data Bank (NTDB), and the NTDB National Sample Project (NSP), with the latter reporting nearly 25% missing data. Despite being the most commonly used tool for benchmarking trauma outcomes, TRISS has important limitations that could account for such a wide difference in survival for patients sustaining critical injuries, especially those more than 55 years old [34, 35].

## Conclusions

Our findings show anatomical injury distributions and mortality rates among three age groups of patients involved in motorcycle trauma. The age of the patient is a predictor of death, and patients older than 55 years showed an increased mortality rate, more commonly secondary to chest injuries as compared with younger patients. This finding could suggest the necessity of a more aggressive treatment for this subset of patients. Our study further strengthens the importance of utilizing a level I trauma center in the management of these patients as demonstrated by their improved survival rates.

## Data Availability

The datasets generated and/or analyzed during the current study are available from the corresponding author on reasonable request.

## Abbreviations

AIS: Abbreviated Injury Scale
ASA: American Society of Anesthesiologists
CI: Confidence interval
ISS: Injury Severity Score
NSP: National Sample Project
NTDB: National Trauma Data Bank
OR: Odds ratio
PS: Probability of death
RTS: Revised Trauma Score
TRISS: Trauma and Injury Severity Score
